# Terminal bifurcation and unusual communication of left testicular vein with the left suprarenal vein

**DOI:** 10.1590/1677-5449.002017

**Published:** 2017

**Authors:** Satheesha Badagabettu Nayak, Ashwini Aithal Padur, Naveen Kumar, Deepthinath Reghunathan

**Affiliations:** 1 Manipal University, Manipal, India.

**Keywords:** left testicular vein, variation, bifurcation, suprarenal vein, varicocele, veia testicular esquerda, variação, bifurcação, veia suprarrenal, varicocele

## Abstract

Variations of the testicular veins are relevant in clinical cases of varicocele and in other therapeutic and diagnostic procedures. We report herein on a unique variation of the left testicular vein observed in an adult male cadaver. The left testicular vein bifurcated to give rise to left and right branches which terminated by joining the left renal vein. There was also an oblique communication between the two branches of the left testicular vein. A slender communicating vein arose from the left branch of the left testicular vein and ascended upwards in front of the left renal vein and terminated into the left suprarenal vein. The right branch of the testicular vein received an unnamed adipose tributary from the side of the abdominal aorta. Awareness of these venous anomalies can help surgeons accurately ligate abnormal venous communications and avoid iatrogenic injuries and it is important for proper surgical management.

## Resumo

Variações nas veias testiculares têm relevância em casos clínicos de varicocele e em outros procedimentos terapêuticos e diagnósticos. Relatamos aqui uma variação peculiar de veia testicular esquerda observada em um cadáver adulto do sexo masculino. A veia testicular esquerda bifurcava para dar origem aos ramos esquerdo e direito, os quais terminavam juntando-se à veia renal esquerda. Foi observada também comunicação oblíqua entre os dois ramos da veia testicular esquerda. Uma fina veia comunicante originava-se do ramo esquerdo da veia testicular esquerda e ascendia até a frente da veia renal esquerda, terminando na veia suprarrenal esquerda. O ramo direito da veia testicular recebia um tecido adiposo tributário não especificado da lateral da aorta abdominal. Conhecimento dessas anomalias venosas pode ajudar os cirurgiões a ligar corretamente comunicações venosas anormais e evitar danos iatrogênicos e é importante para um manejo cirúrgico apropriado.

## INTRODUCTION

Over recent years, variants of the testicular vessels have become more important because of advances in surgery and invasive interventions such as laparoscopic surgery and kidney transplantation. Venous blood from the testis is drained through the pampiniform plexus of veins. This plexus condenses to form four veins at the superficial inguinal ring; two veins at the deep inguinal ring and one testicular vein at varying levels. The right testicular vein terminates into the inferior vena cava and the left testicular vein terminates into the left renal vein.^1^ Knowledge of variations of the testicular veins is important during clinical management of varicocele and retroperitoneal surgeries. Varicocele is the abnormal dilatation of the pampiniform plexus of veins, which can cause testicular atrophy and is said to affect approximately 15% of the male population.^2^^,^ 3 Adequate knowledge of vascular anomalies of testicular veins will help radiologists and surgeons to recognize and protect these veins which play major roles in the thermo-regulation that is essential for the efficient functioning of testis. Reported variations of the testicular vein include variations in the course, areas of drainage, and termination. Duplication of the vein is one commonly reported anomaly. A duplicated right testicular vein was noted in 4% of specimens in one study^4^ and in 15% of the population in another study.^5^ Bifurcation of the right testicular vein has been reported by Nayak et al.,^6^ associated with an arteriovenous anastomosis. We report a very rare variation of the left testicular vein, having a terminal bifurcation, presence of communicating branches, and unusual communication with the left suprarenal vein. We consider that the variations encountered might reflect a complicated process of embryogenesis in this area and could possibly be a predisposing factor for left sided varicocele. An attempt has therefore been made to explicate the possible embryological basis of this anomalous variation and to emphasize its clinical implications.

## CASE REPORT

**Figure 1:**
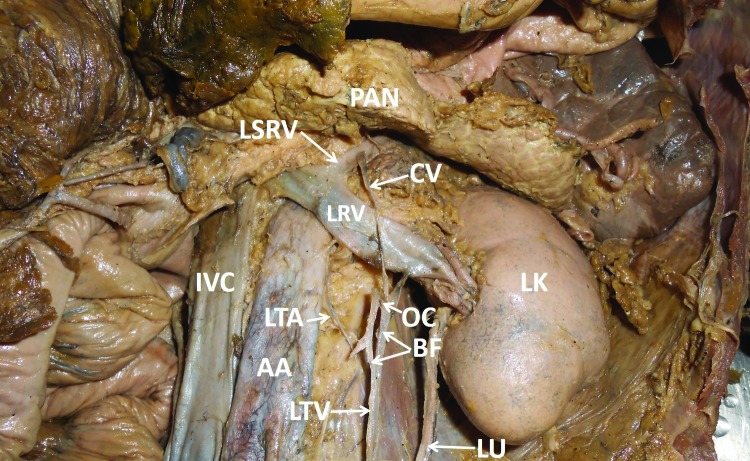
Dissection of the abdomen showing the anatomical variant of the left testicular vein. AA = abdominal aorta; BF = bifurcation of left testicular vein; CV = communicating vein between testicular and suprarenal veins; IVC = inferior vena cava; LK = left kidney; LRV = left renal vein; LSRV = left suprarenal vein; LTA = left testicular artery; LTV = left testicular vein; LU = left urethra; OC = oblique communication between left and right branches of testicular vein; PAN = pancreas.

**Figure 2:**
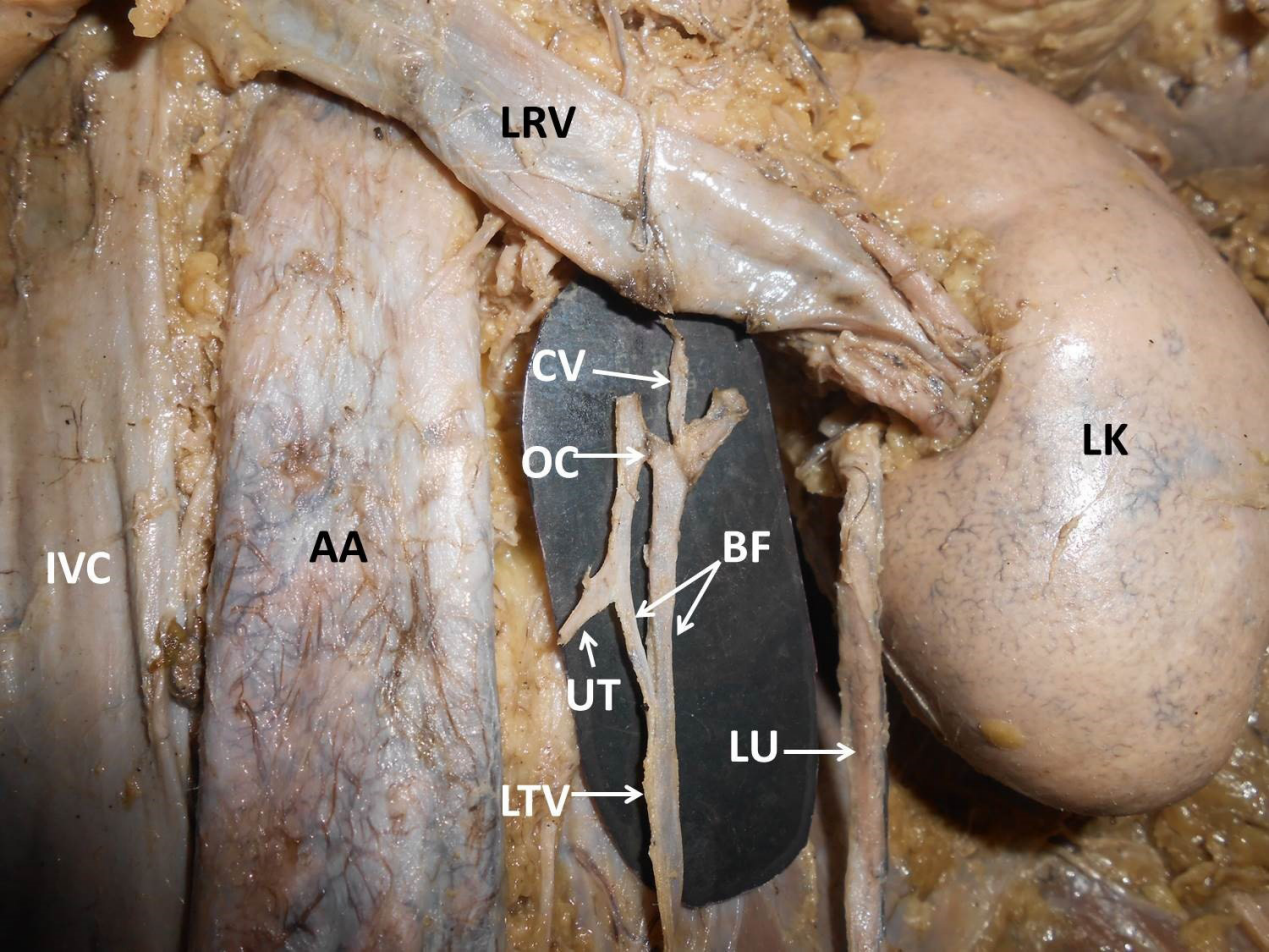
Closer view of the terminal part of the left testicular vein. Terminal part of the testicular vein has been pulled down to show the bifurcation and communications clearly. AA = abdominal aorta; BF = bifurcation of left testicular vein; CV = communicating vein between testicular and suprarenal veins; IVC = inferior vena cava; LK = left kidney; LRV = left renal vein; LTV = left testicular vein; LU = left urethra; OC = oblique communication between left and right branches of testicular vein; UT = unnamed adipose tributary joining the right branch of testicular vein.

During dissection classes for medical undergraduates, we observed a unique variation of the left testicular vein in an adult male cadaver aged approximately 70 years. The left testicular vein was formed by the union of the pampiniform plexus of veins, as described in anatomy textbooks. Its course in the lower part of the abdomen was also normal. It ascended retroperitoneally towards the left renal vein. Approximately 3 cm below the left renal vein, it bifurcated to give rise to left and right branches (Figure 1). The left and right branches terminated by joining the left renal vein. There was an oblique communication between the two branches of the left testicular vein. This communicating vein was about 0.5 cm long and ascended obliquely from the left branch of the testicular vein to join the right branch, 0.5 cm below the left renal vein. Further, there a slender communicating vein arose from the left branch of left testicular vein, 0.5 cm below its union with the left renal vein. This communicating vein ascended upwards and to the right, in front of the left renal vein and terminated into the left suprarenal vein (Figure 2). The right branch of the testicular vein received an unnamed adipose tributary from the side of the abdominal aorta.

## DISCUSSION

The importance of anatomical variants of the testicular veins has greatly increased in recent times because of the development of advanced operative procedures within the abdominal cavity for varicocele and undescended testes.^7^ These variations also have practical importance in renal transplantation, renal and gonadal surgeries, uroradiology, gonadal or testicular color Doppler imaging, spermatic venography, and other retroperitoneal therapeutic and diagnostic procedures.^8^ Numerical variations, atypical course and unusual drainage of the testicular veins are attributed to their embryologic origin. Embryogenesis of the inferior vena cava involves development, regression, anastomosis, and replacement of three pairs of venous channels: the posterior cardinal, the subcardinal, and the supracardinal veins. Bilateral anastomoses between the supracardinal and subcardinal veins form the renal segment of the inferior vena cava (IVC).^9^ When this communication has been established, the left subcardinal vein disappears, with only its distal portion remaining, as the left testicular vein.^10^ The terminal bifurcation of the left testicular vein observed in the present case might be due to bifurcation of the left subcardinal vein. The left suprarenal vein also arises from the left subcardinal vein. The unique communication between the left testicular vein and left suprarenal vein seen in the present case might therefore be due to incomplete regression of the left subcardinal vein, during the seventh to eighth weeks of embryogenesis.

Testicular vein variations have been extensively studied by Asala et al.,^11^ who were of the opinion that variations of the left testicular vein are more common than on the right, since they found variations in 21.3% of the specimens they studied.^11^ The presence of three left testicular veins has been reported in the literature^12^ with incidence estimated to be approximately 1 to 2%.^4^ A case of four left testicular veins with an incidence of 1% has also been reported.^4^ However, terminal bifurcation of the left testicular vein is a very rare variation. Drainage of the left testicular vein into the IVC,^13^ left suprarenal vein,^14^ and left subcostal vein^15^ have also been reported. In our case, the bifurcated left testicular vein drained into the left renal vein, but there was an unusual communication between the right and left branches of the bifurcated vein and a communicating vein connecting the bifurcated vein with the left suprarenal vein was also present. Additionally, the right branch of the testicular vein received an unnamed adipose tributary from the side of the abdominal aorta. Because of these multiple variations, the area around the renal hilum appeared more complex than normal. Presence of these variations might cause confusion when evaluating radiological findings or during surgeries related to the kidneys and suprarenal glands.

Varicocele is a well-recognized cause of decreased testicular function and is said to be present in about 40% of infertile males.^16^ Varicocele is more common on the left than the right due to the course of the vein behind the sigmoid colon and the perpendicular relationship of the left testicular vein in relation to the left renal vein. The bifurcated vein in the current case could cause stasis in the left pampiniform plexus and further increase the possibility of varicocele. The incidence of varicocele recurrence after surgery varies from 0.6 to 45%.^17^ One of the factors affecting recurrence of varicocele is variant anatomical distribution of the testicular veins. Surgical occlusion of the testicular veins, particularly the left testicular vein, is the most popular treatment for varicocele.^18^ It is therefore imperative for surgeons and andrologists to be aware of such anomalous variations for accurate diagnosis and proper surgical planning. Additionally, this unusual bifurcation of the left testicular vein with abnormal communications could lead to a condition which has rarely been described in the literature, known as testicular vein compression syndrome; in which the vein is enlarged and usually thrombosed. This could result in hydronephrosis secondary to compression of the adjacent ureter.^19^ Urologists should therefore bear in mind the possibility of this type of variation in cases of obstructive uropathy, especially upper urinary tract dilatation.

## CONCLUSION

The variations we report here are surgically very important because they could go unnoticed until discovered during surgery. The communicating vein could suffer iatrogenic injuries during suprarenal surgery, leading to bleeding. The terminal bifurcation of the testicular vein could also lead to increased chances of its damage during upper abdominal retroperitoneal surgeries. One of the segments of the bifurcated vein could be used as a graft during surgeries. Left-sided varicocele is more common than right. The variant bifurcated termination of the left testicular vein and its communication with the left suprarenal vein could further increase the possibility of a left-sided varicocele. It could also result in post-surgical recurrence of varicocele. In view of the practical importance of such variations to renal transplantation, renal and gonadal surgeries, and other therapeutic and diagnostic procedures, the present report is of significant importance to surgeons, radiologists and andrologists.
